# Achilles tendon rupture following surgical management for tendinopathy: a case report

**DOI:** 10.1186/1471-2474-8-19

**Published:** 2007-02-27

**Authors:** Michael R Carmont, Nicola Maffulli

**Affiliations:** 1 Department of Trauma & Orthopaedic Surgery, University Hospital of North Staffordshire, Keele University School of Medicine, Stoke on Trent, ST4 6QG, UK

## Abstract

**Background:**

Achilles tendinopathy is understood to be a failed healing response. Operative management is utilised following the failure of non-operative methods.

**Case Presentation:**

We present a case of Achilles tendon rupture, sustained whilst isometrically loading the Achilles tendon during an eccentric loading exercise programme. Conclusion: Bilateral surgical exploration and debridement had previously been performed after conservative management of bilateral Achilles tendinopathy had been unsuccessful.

## Background

Achilles tendinopathy is understood to be a failed healing response process involving separation of collagen bundles, increase of hydrophilic extracellular matrix, haphazard neovascularisation, and absence of inflammatory cells [1,2]. Patients present with pain and thickening of the tendon [3]. Tendinopathy may lead to reduced tensile strength and a predisposition to rupture [4]. Management aims to alleviate symptoms and reduce the theoretical risk of rupture. Conservative methods include eccentric loading exercises and the avoidance of aetiological factors [5]. Operative management is utilised following the failure of non-operative methods [6,7].

We report the case of a patient who experienced an Achilles tendon rupture following surgical exploration of Achilles tendinopathy.

## Case Presentation

A 44 year old retired Police Officer had a 4 year history of bilateral Achilles tendinopathy. The ailment had been managed conservatively with eccentric loading exercises and the avoidance of aetiological factors [5], but his symptoms failed to settle. The patient underwent surgical exploration to both tendons using a medial approach. The paratendon was excised, and fish mouth fasciotomies of the crural fascia were performed proximally [8]. Small similarly sized nodules were present in both tendons. Longitudinal tenotomies were performed through the nodules within the tendons, and the tendinopathic areas were excised. The fat from Kager's triangle was also detached from the anterior aspect of the tendon [8]. Post-operatively, the patient was immobilised in a below knee synthetic cast, fully weight bearing as able. The casts were removed two weeks after surgery, and routine rehabilitation consisting of 3–4 episodes of eccentric loading exercises as limited by pain, was commenced [9]. At five weeks following the operation, whilst standing on his toes, he experienced a sharp pain to his left Achilles tendon, and he was subsequently unable to weight bear.

**Figure 1:**
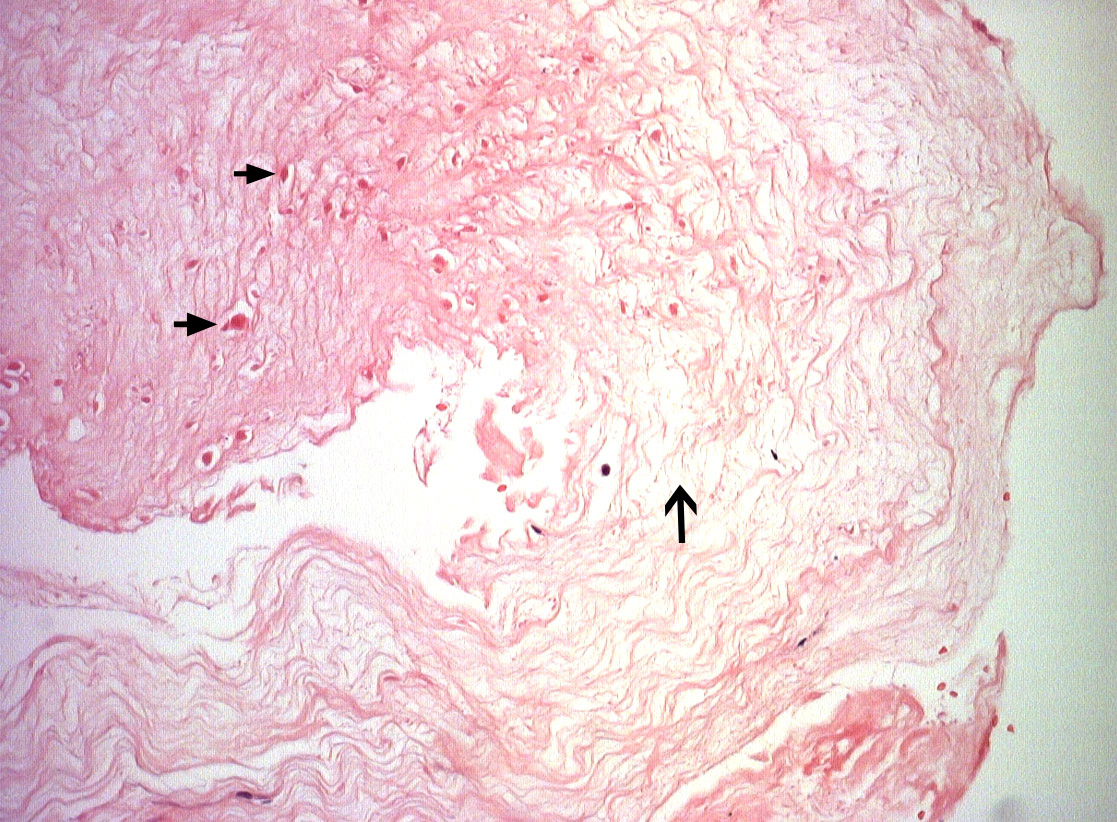
Histology of the tendinopathic tendon at repair (Transverse plane, × 20 Magnification). Revealing scanty hypocellular degenerate tendon displaying separation of collagen fibrils (solid arrows) and disorganisation. Small pieces of fibrin (hollow arrow) were also present and there was no inflammation or neovascularisation.

On examination the day after this episode, Simmond's [10] and Matles's [11] tests suggested an acute rupture of his Achilles tendon. The leg was placed in a below knee synthetic cast with the ankle in equinus, and the tendon was repaired percutaneously [12]. Complete rupture of the tendon was noted at operation. Histology revealed findings consistent with Achilles tendinopathy although some of these changes may be secondary to initial surgery (Figure 1). Post-operative recovery was uneventful, and the patient recovered his full function.

## Conclusion

Tendon healing occurs in three overlapping phases [3]. The initial phase lasts 24 hours, and involves an intense inflammatory response with neutrophils, monocytes and macrophages. Vasoactive and chemotactic mediators stimulate vascular permeability, angiogenesis and tenocyte proliferation with type III collagen being produced. The second proliferative phase commences after a few days, with peak synthesis of type III collagen. After approximately six weeks, the remodelling phase commences, with decreased cellularity and decreased collagen and glycosaminoglycan synthesis. Consolidation occurs, and the repair tissue becomes more fibrous and the collagen fibres become aligned according to the direction of stress, and a greater proportion of type I collagen is synthesised. Eventually over the course of twelve months, during the maturation stage of healing, the fibrous repair matures into scar tissue [3].

Stretching is likely to increase collagen synthesis and improve fibre alignment leading to an increased tensile strength during healing. This should occur once the inflammatory phase of healing has settled [13]. Eccentric loading has been shown to produce better outcomes than with concentric loading exercises [14,15]. These exercise programmes have been shown to reduce pain and improve function, although their effectiveness in the general population has recently been considered [16]. Ultrasound scanning shows reduced tendon thickness and intratendinous signal [17], which can normalise following successful management [18].

Eccentric loading exercises form the basis of non-operative management, and most patients will respond if recognised early. In patients with tendinopathy recalcitrant to non-operative measures, surgical exploration leads to good results [19-22]. We appreciate that there are several surgical methods, that it is not known which is the best way to remove degenerate tissue and that prolonged recovery following surgery may occur. Surgery removes adhesions and degenerate areas and other factors that influence local circulation [23].

No management is without complication, and these procedures carry a post-operative complication rate of up to 11% [24]. However, we believe that Achilles tendon rupture following surgical exploration for tendinopathy has not been previously reported [25]. At post-operative rehabilitation, the patient was attempting to stand on tip toes from a midstance position, thus performing an isometric loading exercise when his tendon ruptured. This was at five weeks following surgery at the end of the proliferative phase of tendon healing. Eccentric exercises are commonly performed in the rehabilitation programme following surgery for Achilles tendinopathy, they are considered safe, and we do not know why they resulted in a rupture in this particular patient. We appreciate that it may be difficult for a patient to recount the exact position and movement of the ankle at the time of rupture, our patient had already undertaken, all be it unsuccessfully, an eccentric exercise programme for his tendinopathies. Also, we cannot explain why only one tendon was affected, as both tendons were the same to visual inspection, underwent the same surgical procedure, and the same post-operative management regimen.

## Competing interests

The author(s) declare that they have no competing interests.

## Authors' contributions

MC wrote the case report including performing the literature review. NM is an experienced Trauma & Orthopaedic Surgeon with an interest in the Achilles tendon. NM provided guidance for the literature search, the writing of the paper and also proof read the paper. Both authors have read and approved the final manuscript.

## Pre-publication history

The pre-publication history for this paper can be accessed here:
